# Drainage failure and associated urban impacts under combined sea-level rise and precipitation scenarios

**DOI:** 10.1038/s41598-025-07332-8

**Published:** 2025-07-02

**Authors:** Chloe Obara, Charles H. Fletcher, Shellie Habel, Kristian McDonald, Kayla Yamamoto

**Affiliations:** 1 Department of Earth and Planetary Sciences, School of Ocean and Earth Science and Technology, University of Hawaiʻi at Mānoa, POST Building, 1680 East-West Road, Honolulu, HI 96822 USA; 2 Forerunner Industries, Inc, 548 Market St #93531, San Francisco, CA 94104-5401 USA

**Keywords:** Sea-level rise, Compound flooding, Drainage failure, PCSWMM, Corrosion, Environmental sciences, Hydrology

## Abstract

Existing sea-level rise models for coastal cities often neglect precipitation impacts on infrastructure. In tidally influenced areas, high water levels can overwhelm stormwater systems, causing drainage failure, corrosion, and backflow of contaminated water. Waikīkī, Honolulu’s tourism hub, faces increasing flood risks and infrastructure damage due to rising sea levels. Using PCSWMM modeling software, selected for its capacity to represent complex urban drainage systems, this study simulates drainage failure under present and projected sea levels with precipitation. Findings reveal a 5-year precipitation event at present sea level floods more inlets than three feet of sea-level rise, while a 10-year event floods three times more inlets than four feet of sea-level rise. By 2050, a 5-year event could disrupt transportation and contaminate 70% of stormwater inlets in Waikīkī. Accounting for precipitation, 100% of outfalls will fail and 85% of the drainage system will be full by 2040. Results indicate 22–50% more flooded inlets during precipitation events than passive models at present sea level. Salinity and water level data indicate severe corrosion risks, potentially worsening drainage failure. This study highlights the urgent need to integrate precipitation into sea-level rise modeling to strategically mitigate urban flood risks in Waikīkī and other coastal cities.

## Introduction

Coastal regions less than 10 m above sea level account for just 2% of the Earth’s land area, yet accommodate 13% of the global urban population^1,2^. Estimates by the Organisation for Economic Co-operation and Development indicate that by 2070, around 150 million people living in these coastal areas could face flooding risks due to sea-level rise, increased storm intensity, land subsidence, population growth, and urban development^3^. When flood risks occur simultaneously or consecutively, volume and extent of flooding can be heightened, a state known as compound flooding^4^. Climate-driven processes contributing to compound flood events include long-term forcings such as sea-level rise (SLR) and elevated groundwater, as well as short-term events such as extreme high tides (colloquially referred to as king tides) and precipitation events^5–7^. The IPCC 6th Assessment Report concludes, with high confidence, that sea levels will continue to rise for centuries to millennia, with observations already surpassing the projected low and intermediate-low scenarios^8,9^. Furthermore, uncertainties in the melting rates of Antarctic glaciers may be leading to a drastic underestimation of SLR rates^10,11^. Projections also alert to a global trend of less frequent but more severe precipitation^8^. The potential economic repercussions of climate extremes on coastal cities are staggering, estimated at approximately $35 trillion and affecting assets such as buildings, transportation networks, utility infrastructure, and ports by 2070^3^.

### Sea level and precipitation trends

The relative sea level trend for Honolulu, based on monthly mean sea level data from 1905 to 2023 at the NOAA Honolulu tide station (station ID: 1,612,340)^12^, is an increase of 1.54 ± 0.2 mm per year (95% confidence interval). However, monthly linear trend data at this station shows that, over the past 30 years, the rate has increased to 3.65 ± 0.25 mm per year (95% confidence interval). While SLR is relatively gradual, monthly tidal extremes offer insights into conditions associated with projected higher sea levels^13^. Nuisance flooding occurs when tides exceed 0.35 m above the mean higher high water (MHHW) datum^14^. Over the past 10 years, the average king tide in Honolulu has elevated water levels by 0.33 m above MHHW, which is equivalent to one foot of SLR. As sea levels rise, high tides will more frequently exceed the nuisance flood threshold, projected to increase from 2 days per year to 63 days per year before 2050 under the NOAA intermediate SLR scenario^15^.

Annual precipitation in Hawaiʻi has decreased since the early 1900s, while long-term trends in the frequency and intensity of tropical cyclones in the Pacific region remain relatively stable^16–18^. However, increased variability in precipitation in the eastern tropical Pacific due to global warming places Hawaiʻi at greater risk of periodic heavy precipitation, more powerful El Niños, and the co-occurrence of weather systems such as monsoon precipitation and hurricanes^16,19–22^. Despite the uncertainty in long-term precipitation projections for Hawaiʻi, modern storm intensity data can be useful in representing realistic scenarios, with the caveat that they may represent a conservative estimate of future conditions.

### Storm drainage infrastructure vulnerability

A hidden effect of SLR that may be going unnoticed is the subterranean impact on coastal infrastructure^23^. Gravity-flow stormwater drainage systems are common in coastal urban municipalities worldwide and are highly vulnerable to the effects of SLR^24–27^. The functionality of these drainage systems relies on an elevation gradient between the water level at the higher inlets and the lower outflow points. In low-elevation coastal regions, high tides and rising sea levels can reduce this gradient, slowing or reversing the drainage process^28^. Furthermore, numerous studies show that the occurrence of seawater and wetting–drying conditions in and surrounding storm drainage infrastructure results in higher corrosive potential for concrete and steel rebar^29–32^. Corrosion and cracking of concrete pipes has been documented in coastal cities such as Melbourne, Australia and Orlando, Florida^33,34^. In Honolulu and San Diego, cracks in storm drainage piping have caused sinkholes, affecting residents and businesses^35,36^. Furthermore, tracers of urban runoff including fecal contamination have been documented in coastal storm drainage systems during high tides and precipitation events in North Carolina^37,38^. Stormwater infrastructure is typically designed based on historical data that assumes an unvarying probability of extreme precipitation. However, this approach is becoming increasingly outdated as climate change alters the frequency and intensity of precipitation events, leading to a higher likelihood of extreme precipitation that exceeds past norms ^8,39,40^. Therefore, drainage infrastructure designed using mid-twentieth century precipitation records may be subject to precipitation conditions that differ from existing design standards^41^.

### Study area

**Fig. 1:**
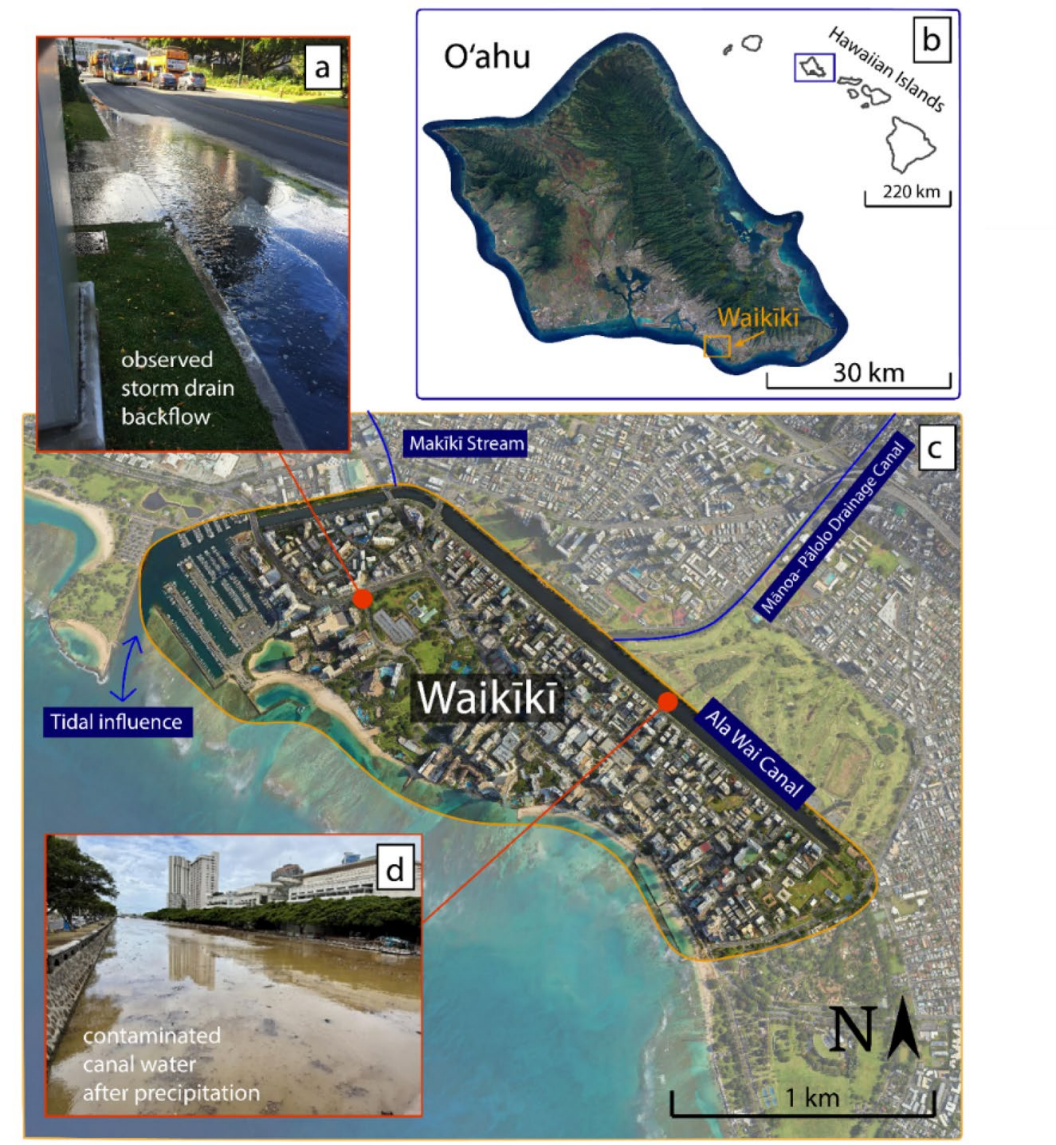
Waikīkī (**c**) is located on the southeast coast of the island of Oʻahu, part of the Hawaiian Island chain (**b**). Inset images show an example of sunny day backflow occurring during a King Tide in 2017 (**a**; credit, Hawaiʻi and Pacific Islands King Tides Project, University of Hawaiʻi Sea Grant, 2024) and an example of contaminated water in the Ala Wai Canal after a precipitation event (**d**; credit, Michael Cain). The software used to generate the map in this figure is Google Earth Pro version 7.3 https://earth.google.com/, and Adobe Illustrator version 28 https://www.adobe.com/products/illustrator.html.

This study was conducted in the Waikīkī area of Honolulu on the island of Oʻahu, Hawaiʻi, a highly urbanized, low-lying coastal area and the state’s tourism hub, located along the Pacific Ocean (Fig. 1). With 97% of its ground surface less than 3 m above local mean sea level and 88% of its geographic boundary influenced by tides, Waikīkī is highly vulnerable to flooding. Addressing this vulnerability is crucial, as the U.S. Army Corps of Engineers estimates that a major flooding disaster could damage over 3,000 structures and result in nearly $1 billion (2023 dollars) in structural damages^42^. Such damages would affect 42% of the state’s visitor industry revenue and 8% ($5 billion) of the Gross State Product^43^.

Like many urban areas, Waikīkī has undergone extensive urban hardening, with 78% of its total land area covered by impervious surfaces, limiting natural drainage. To address this issue, about 20 km of gravity-driven drainage pipes have been installed to route storm water to 62 outfalls. Over 75% of the storm drainage system discharges landward into the Ala Wai Canal, a 3.1 km long manmade channel with a single direct connection to the open ocean via the Ala Wai Boat Harbor. The canal serves as the outlet of the Mānoa-Pālolo Drainage Canal and Makīkī Stream, which drain a 48.75 km^2^ watershed, making it comparable to a small-scale estuary influenced by both tides and precipitation (Fig. 1,^44^). With only one outlet to the ocean and extensive urban runoff inputs, the canal’s water has been found to contain high concentrations of pathogens such as *V. parahaemolyticus*, posing a public health hazard if this water backflows into the streets^45,46^. At least one documented fatality has occurred following contact with canal water^47^. As the presence of an urban drainage canal or estuarine feature is common for coastal municipalities, these findings are relevant to many coastal urban areas, such as New York City, Boston, San Francisco, Seattle, Philadelphia, London, Shanghai, and Sydney.

Factors such as system lifespan, pipe corrosion, proximity to coastline, and shallow groundwater depth may be contributing to the reduced functionality of the Waikīkī storm drainage system^48^. According to the Honolulu Department of Planning and Permitting, 70% of the Waikīkī drainage system is made of reinforced concrete^49^. As-built maps of the storm drainage system in Honolulu date back to 1916. Since the projected lifespan of stormwater systems made of reinforced concrete is typically 100 years, much of the original storm drainage infrastructure in Waikīkī is likely at high risk of defects^50,51^. Tropical areas like Hawaiʻi also experience higher corrosion rates than those in temperate climates due to higher sustained temperatures^32^.

Presently, there are insufficient resources for the City and County of Honolulu to routinely monitor drain lines for defects, making the full extent of the issue unknown. However, infrastructure failures are already being observed during high tides, leading to stormwater drainage backflow, traffic slowdowns due to flooded roadways, and partial inundation of active cesspools^52,53^.

**Fig. 2:**
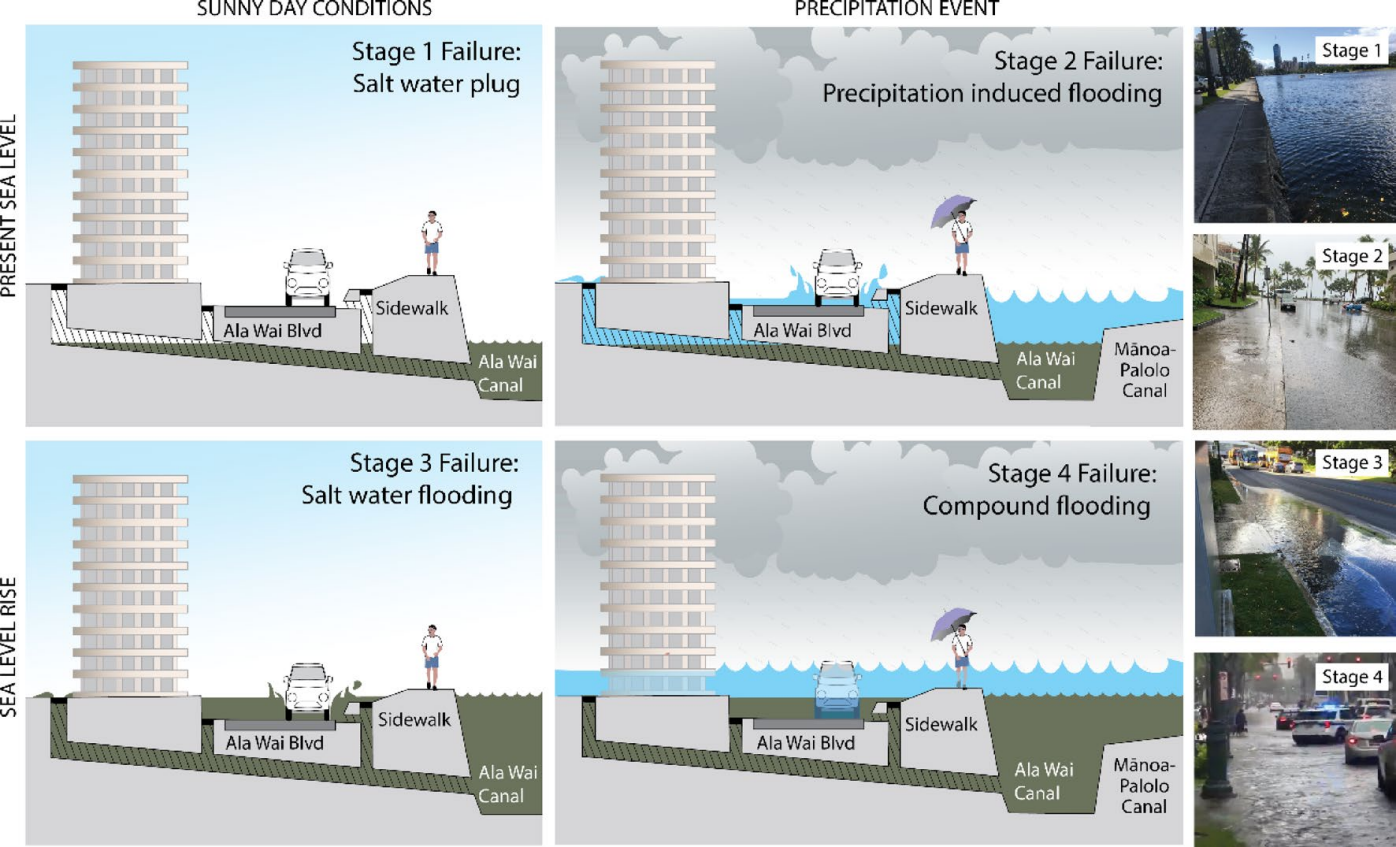
Stages of failure visualized for the Waikīkī storm drainage system. Adaptation from Evans and Zelenski (2019) by Nancy Hulbirt. Images on the right show observed examples of each stage occurring in Waikīkī. Image credit, Chloë Obara and Hawaiʻi and Pacific Islands King Tides Project.

Sea-level rise can impact storm drainage system performance well before backflow is observed at inlets^54^. Under normal conditions, the water level remains below the top of the outfall, allowing water to drain freely, and pipes have capacity to effectively route stormwater to outfalls during precipitation events. Figure 2 illustrates drainage failure in four stages:

- *Stage 1:* The water height exceeds the outfall height, creating a plugging effect that may slow drainage.
- *Stage 2:* Precipitation inputs exceed drainage capacity, causing backflow; pipes are completely full.
- *Stage 3:* Sea levels rise high enough to induce saltwater backflow during sunny day conditions.
- *Stage 4:* There is no capacity for drainage and compound flooding occurs, driven by both precipitation and high sea levels.

### Previous work

Understanding the impact of compound flooding on urban areas has been the focus of numerous studies in recent years. It is useful to focus on both discrete and compound flood drivers to better understand complex flood conditions (see reviews in ^55–59^). For example, some studies focus solely on high-tide flooding without considering precipitation^60,61^, while others consider precipitation but not SLR^62–66^. It is also important to consider the combined probability of flood drivers coinciding to generate compound effects^6,7,67,68^. This study integrates both discrete and compound scenarios across multiple flood drivers by analyzing storm drainage performance under scenarios of sea-level rise and precipitation both in isolation and in tandem.

Various hydrologic and hydraulic modeling software tools are available to assess flood scenarios^69^. A model review by Son et al. (2021) found that of fourteen flood models, only four (including the model produced by the study) were capable of accounting for a stormwater drainage system. Models such as ADCIRC, Delft3D and HEC-RAS 2D, while robust in generating flood maps, lack the capability to quantify water level in pipes or flood depths produced at inlets^57^. As the commercial software PCSWMM provides a user-friendly interface and can represent a storm drainage system with a high level of detail at an unlimited system size, this model was selected for this study.

Qiang et al.^70^ demonstrate the importance of considering drainage backflow at outfalls under conditions of low-intensity precipitation and high sea levels. The hydrostatic flood modeling method has been used to approximate drainage failure, assuming water level elevations within the drainage system are uniform and equal to the simulated local sea level^52,61^. This method may underestimate the extent of drainage failure, as it does not account for groundwater inundation, the influence of precipitation, or the dynamic effects of drainage system dimensions. In Honolulu, Habel et al.^52^ evaluated the effects of direct marine flooding, storm drainage backflow, and groundwater inundation on coastal urban areas, emphasizing the need to reassess primary sources of SLR-induced flooding for more effective flood management strategies. This analysis addresses this gap by accounting for groundwater, precipitation, and storm drainage dimensions, and comparing results to existing hydrostatic models to better understand the influence of these unconsidered variables on urban flooding.

Observational data is a crucial component of a robust model validation process. Brendel et al.^63^ illustrate this by developing a 1D/2D method for simulating present-day flow depths in storm drainage infrastructure in Roanoke, VA, and validating their results with observed storm characteristics. Sangsefidi et al.^55^ assess the vulnerability of coastal storm drainage systems to compound seawater, groundwater inundation, and stormwater flooding in Imperial Beach, CA, validating their results with observational data from the storm drainage system. Shi et al.^64^ use a coupled 1D/2D model to quantify the influences of coastal dams, drainage systems, tide gates, rain, and storm surges on compound flooding in Xiangshan, China, validating their model with observed river gage data. Tang et al.^71^ use Delft3D to evaluate the combined impacts of precipitation and sea level in Sunset Beach, CA, incorporating hydraulic infrastructure and validating their model with water level measurements and photographic reconstructions. Gold et al.^72^ find that roadway flood frequency along the US Atlantic coast can be higher than suggested by tidal data, highlighting the importance of instrumenting storm drains to capture additional flood drivers. Thelen et al.^73^ use water level sensors to validate flood projections for Carolina Beach, NC, using a robust coupled model that accounts for the compounding effects of tides, winds, and rain. Although these studies confirm the vulnerability of coastal urban areas to flooding events, additional research is needed to better understand drainage infrastructure vulnerability at resolution useful for local planning.

### Study objectives

In this study, we employ a robust modeling approach using the hydraulic and hydrologic model Personal Computer Storm Water Management Model (PCSWMM) to simulate drainage failure and evaluate the effects of SLR and precipitation on a coastal urban storm drainage system outflowing to a tidally-influenced fluvial drainage canal^74^. We validate our methodology using water level data collected via pressure transducers in the Waikīkī storm drainage system. Lastly, we investigate trends in salinity and temperature based on a one-year dataset of monitoring water at discrete storm drainage locations*.*

This study utilizes sea level benchmarks and design storms for optimal relevance in urban planning. As urban infrastructure planning should account for maximum capacity limitation on the storm drainage system, we use the highest point in the tidal cycle (MHHW) as our condition for present sea level. The City and County of Honolulu Climate Change Commission advises using the intermediate high SLR scenario for all public infrastructure planning and design projects^75^. To produce results useful at a timeline for both near-term (15–20 years) and long-range (20–50 + years) urban planning, we run scenarios applying SLR in one-foot increments up to 4 feet, which, under the intermediate-high projection, is expected to be reached by the year 2080^76^. The U.S. Army Corps of Engineers is presently conducting a flood risk management study for the Ala Wai watershed^42^. The primary objective of the proposed designs is to reduce risks associated with a 100-year, 24-h precipitation event. To align with this goal, we apply the same precipitation event to our sea level scenarios. We also selected the 5-year event to simulate more frequently expected flooding conditions. Finally, we selected the 10-year event because storm drainage systems in Honolulu are designed to handle a 10-year, 24-h storm, so this simulated condition is especially relevant for urban infrastructure planning and development.

## Results

### 1D mapping analysis

**Fig. 3:**
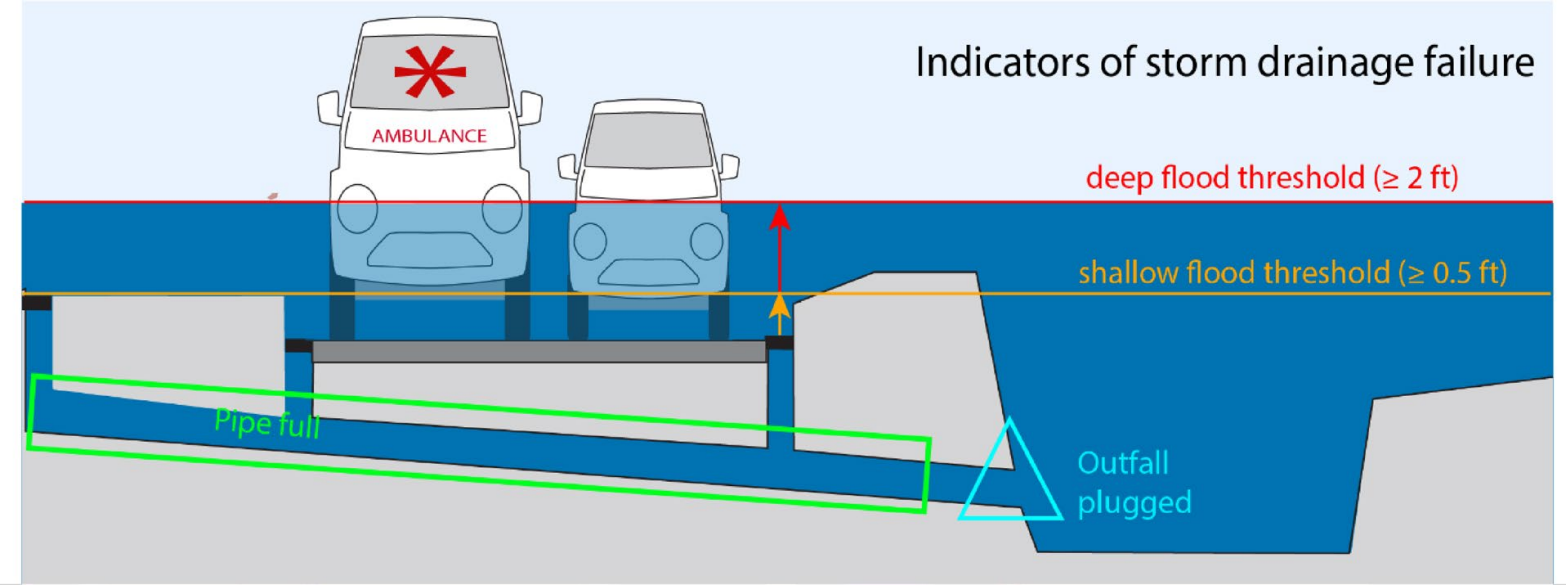
Drainage failure indicators in Waikīkī. Plugged outfall is indicated in light blue, full pipe is boxed in green, shallow flood threshold of equal to or greater than 0.5 feet above the inlet surface is denoted in orange, and the deep flood threshold of equal to or greater than 2 feet above the inlet surface is denoted in red.

Drainage system capacity was evaluated considering the functionality of inlets, pipes, and outfalls at modeled increments of SLR both with and without a precipitation event. To conduct this analysis, we selected measurable indicators for inlets, outfalls, and pipes (Fig. 3). When water height exceeds the surface height of an outfall, we consider the outfall to be ‘plugged,’ in alignment with stage 1 failure. This can inhibit gravity-driven drainage, which relies on an elevation gradient between the water level at the inlet and the outflow points. Plugged outfalls indicate water is backing up into the drainage system. When water level in a pipe reaches the top of its upstream and downstream ends, the pipe is full and has no capacity to accommodate stormwater, a condition first reached at stage 2 failure. Finally, when water height exceeds the surface height of an inlet, we consider the inlet to be ‘flooded.’ To quantify inlet flooding during present sea level with precipitation (stage 2 failure), sunny day conditions (stage 3 failure), and future sea level with precipitation (stage 4 failure), we chose two depths at which street flooding would occur, presenting hazardous driving conditions. In waters 0.5 ft or deeper (shallow flood threshold), small vehicles can stall, and in waters 2 ft or deeper (deep flood threshold), four-wheel drive vehicles, including emergency vehicles, can stall^77^. Though flood depths less than the shallow threshold can present hazards such as human contact with contaminated water, we chose to focus on the hazard of compromised transportation for our analysis. We also consider the percentage of outlets plugged, pipes full, and inlets flooded that have direct connection to the contaminated Ala Wai canal or the open ocean.

### SLR up to 4 ft; no precipitation

**Fig. 4:**
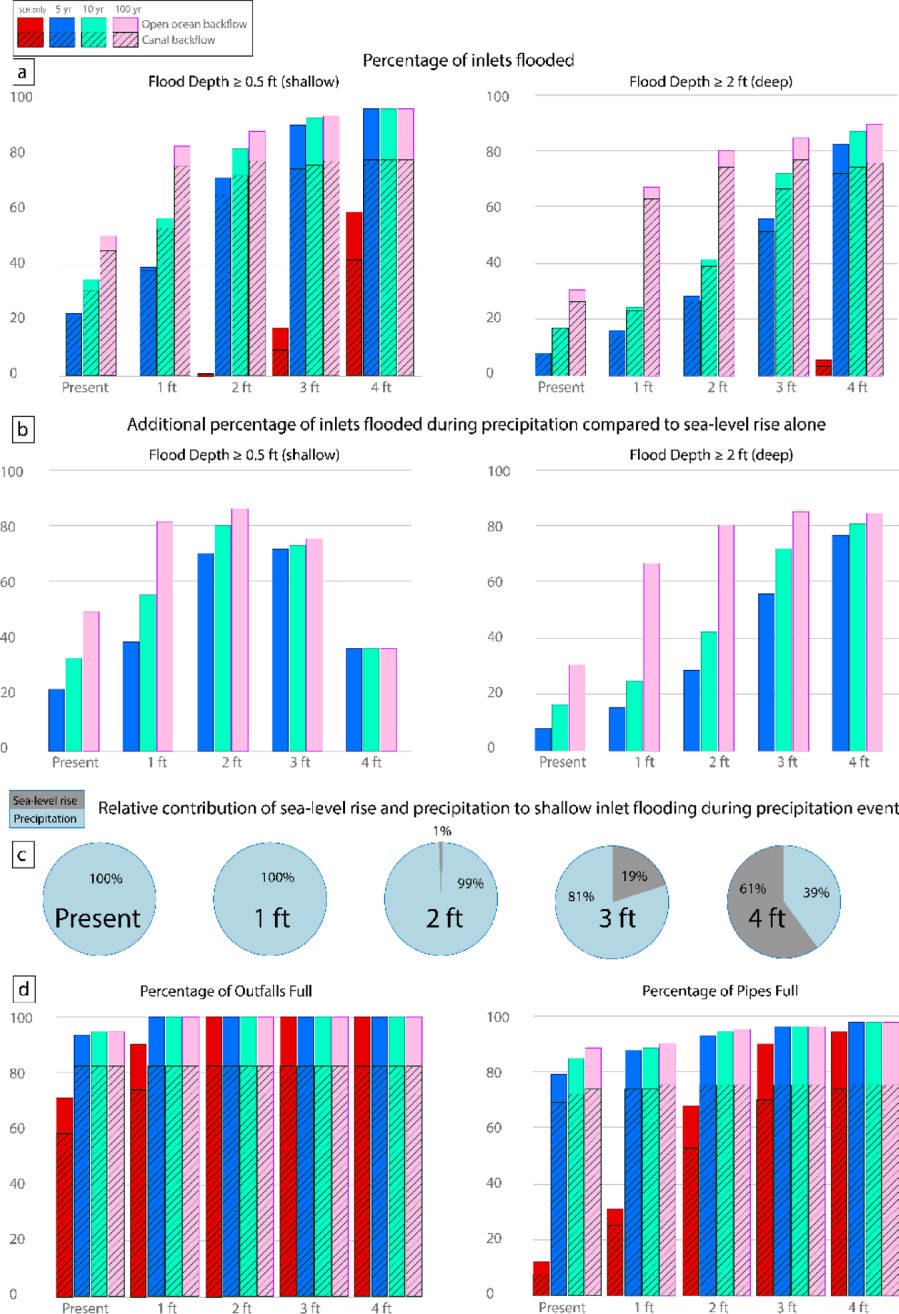
Indicators of failure for the Waikīkī storm drainage system for sea level and precipitation scenarios. Row (**a**) shows percentage of total inlets that reach the shallow (≥ 0.5 ft) or deep (≥ 2 ft) flooding threshold for each scenario. Black shading covers the portion that are connected to the Ala Wai Canal and thus have potential to backflow contaminated water. Row (**b**) shows the additional percentage of inlets flooded by precipitation that otherwise would not be flooded during sunny day conditions (calculated by subtracting the number of inlets flooded by sea level from the number of inlets flooded by precipitation). Row (**c**) shows the relative contribution of sea-level rise and precipitation to shallow flooding during a precipitation event (calculated by dividing the number of inlets flooded at the shallow threshold by precipitation by the total number of inlets flooded at the shallow threshold). The relative contribution remained constant across every precipitation event for a given sea level scenario. Row (**d**) shows percentage of total outfalls and pipes that are full for each scenario, with black shading covering portion connected to the Ala Wai Canal.

**Fig. 5:**
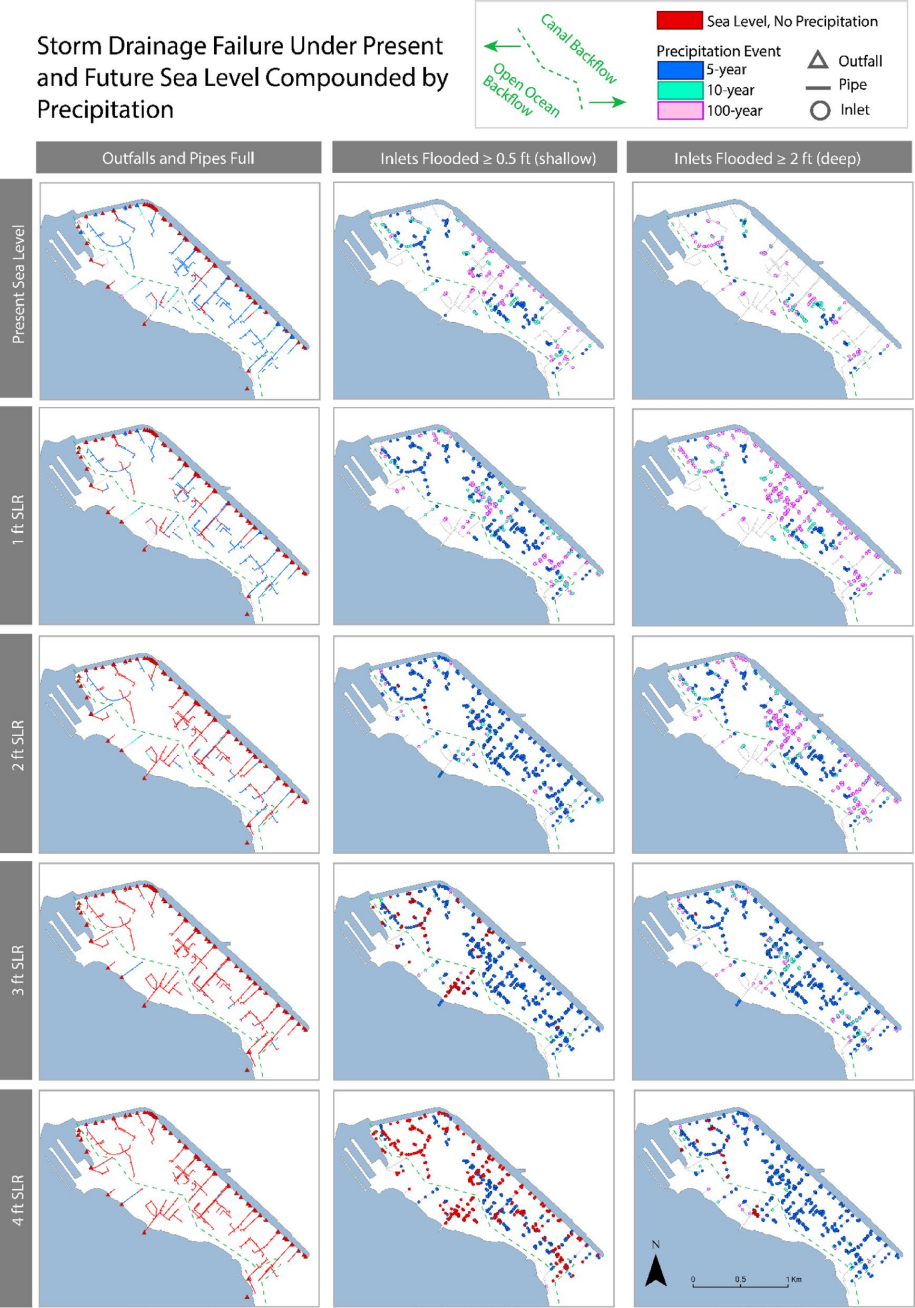
Performance of the Waikīkī storm drainage system under sea level and precipitation scenarios. In the left column, pipes and outfalls are color-coded by the scenario at which they first reach capacity (pipes: full at both ends; outfalls: water above rim elevation). The middle and right columns show inlets color-coded by the scenario at which they first reach shallow (≥ 0.5 ft) or deep (≥ 2 ft) flooding above rim elevation. Present sea level is defined as mean higher high water. The dashed green line denotes the drainage system’s direct connection to the Ala Wai Canal or open ocean, indicating respective backflow sources.

Notably, even at present sea level (MHHW) with no precipitation, over two-thirds of outfall rim elevations are below water, or ‘plugged’ (Figs. 4, 5; Table 1). At and beyond 2 ft SLR, all outfall rims are below water. Over 80% of outfalls at failure have direct connection to the Ala Wai Canal. Percentage of pipes that are full increases from 13% of the pipe system at present sea level to 95% at 4 ft SLR. Percentage of the total pipe system that is both full and has direct connection to the Ala Wai Canal increases from 9% at present sea level to 73% at 4 ft SLR. From present sea level to 2 ft SLR, total inlet flooding at the shallow threshold (≥ 0.5 ft) increases minimally, reaching up to 0.5% of inlets flooded at 2 ft SLR. There is a noticeable increase to 17% of inlets with shallow flooding at 3 ft SLR and a significant increase to 58% with shallow flooding at 4 ft SLR (42% with direct connection to the Ala Wai Canal). No inlets reach the deep flood threshold (≥ 2 ft) until the 4 ft SLR scenario, at which 5% of inlets flood at the deep threshold. Notably, these results are conservative projections of flooding, as evidence of backflow has been already documented in western Waikīkī during king tides with water levels equivalent to 1–2 ft SLR which are not captured by this analysis or previous work ^52^. This discrepancy indicates potential influence of hyper-local conditions such as debris blocks, dumping, or runoff from human activities that are not accounted for in modeled scenarios.

**Table 1:** Storm drainage system failure at scenarios of present sea level and sea-level rise combined with a five-year, ten-year, and one-hundred-year precipitation event.

Scenario	% Pipes full	% Outfalls plugged	% Inlets flooded (≥ 0.5 ft)	% Inlets flooded (≥ 2 ft)
Present sea level	13	71	0	0
1 ft SLR	32	90	0	0
2 ft SLR	68	100	1	0
3 ft SLR	90	100	17	0
4 ft SLR	95	100	58	5
Present Sea Level + 5 yr	79	94	22	8
Present Sea Level + 10 yr	84	95	34	17
Present Sea Level + 100 yr	88	95	50	31
1 ft SLR + 5 yr	87	100	39	16
1 ft SLR + 10 yr	88	100	56	25
1 ft SLR + 100 yr	90	100	82	67
2 ft SLR + 5 yr	94	100	71	29
2 ft SLR + 10 yr	95	100	81	42
2 ft SLR + 100 yr	96	100	87	80
3 ft SLR + 5 yr	97	100	90	56
3 ft SLR + 10 yr	97	100	92	72
3 ft SLR + 100 yr	97	100	93	85
4 ft SLR + 5 yr	98	100	95	82
4 ft SLR + 10 yr	98	100	95	87
4 ft SLR + 100 yr	98	100	95	89

Shallow inlet flooding is defined as when water depth is 0.5 ft or more above the inlet surface elevation and deep inlet flooding is defined as when water depth is 2 ft or more above the inlet surface elevation. Outfalls are defined as plugged when the maximum depth of water in the simulation exceeds the depth of the outfall by greater than 5 cm. A pipe is defined as full when the maximum simulated water level in the pipe reaches the depth of the pipe at any point in the simulation. The vertical uncertainty for this analysis is approximately 1 ft (33.31 cm), so the value at each sea level scenario is has a confidence interval bounded by the scenarios ± 1 ft SLR.

### Present sea level with precipitation; no SLR

To assess present day precipitation-induced flooding, we simulate 5-year, 10-year, and 100-year precipitation events at present sea level, MHHW (Figs. 4, 5; Table 1). We find that outfall capacity limitation for each precipitation event falls between that reached by 1–2 ft SLR, while pipe capacity limitation for each event falls between that reached by 2–3 ft SLR. 100% of inlet flooding at present sea level is driven by precipitation. A 5-year precipitation event at present sea level results in more flooded inlets at the shallow threshold than by 3 ft SLR, and more inlets flooded at the deep threshold than by 4 ft SLR. A 10-year precipitation event results in twice as many inlets flooded at the shallow threshold as by 3 ft SLR and over three times more inlets flooded at the deep threshold than by 4 ft SLR. A 100-year precipitation event produces nearly the same number of flooded inlets at the shallow threshold as by 4 ft SLR, and six times more inlets flooded at the deep threshold than by 4 ft SLR.

### Compound case: SLR and precipitation

To assess compound flood scenarios, we simulate 5-year, 10-year, and 100-year precipitation events under 1, 2, 3 and 4 ft SLR (Figs. 4, 5; Table 1). For every scenario, all outfalls are full and 82% of outfalls have direct connection to the Ala Wai Canal. During a 100-year precipitation event at 1 ft SLR, 90% of pipes are full, equivalent to the percentage of pipes that are full at 3 ft SLR with no precipitation. During a 10-year precipitation event at 2 ft SLR, 95% of pipes are full, equivalent to the percentage of pipes that are full at 4 ft SLR with no precipitation. For all precipitation events at 3 and 4 ft SLR, the percentage of pipes that are full exceeds that at 4 ft SLR with no precipitation, nearing 100% system capacity. For every compound scenario, nearly three fourths of the total pipe system is full and has direct connection to the Ala Wai Canal.

A 10-year event at 1 ft SLR produces as much shallow flooding as 4 ft SLR with no precipitation. The 100-year event at 1 ft SLR and all events at 2, 3, and 4 ft SLR produce more shallow flooding than 4 ft SLR with no precipitation. Precipitation events at 3 ft SLR generate over 70% more shallow flooding than by 3 ft SLR with no precipitation. 95% of inlets are flooded at the shallow threshold at 4 ft SLR with precipitation events; 81% of the total system is flooded and has direct connection to the Ala Wai Canal. Precipitation events generate a 37% increase in shallow flooding and over a 75% increase in deep flooding compared to 4 ft SLR with no precipitation. For the 100-yr event at 2 ft SLR and every event at 3 and 4 ft SLR, over half of all inlets are flooded at the deep threshold and have direct connection to the Ala Wai Canal.

As sea level rises, relative contribution of precipitation to inlet flooding changes (Fig. 4). All or nearly all inlet flooding generated at 1–2 ft SLR is due to precipitation. At 3 and 4 ft SLR, the relative contribution of precipitation decreases, accounting for just over 80% of total shallow flooding at 3 ft SLR and just under 40% of total shallow flooding at 4 ft SLR. This aligns with the increase in sea-level rise-driven flooding seen in the SLR-only scenarios. 100% of deep flooding is driven by precipitation at every SLR interval except 4 ft, where 6% of deep flooding is driven by SLR alone and the remaining 94% of deep flooding is driven by precipitation. The scenario that has the greatest shallow flood contribution from precipitation is the 100-year event at 2 ft SLR. The scenario that has the greatest deep flood contribution from precipitation is the 100-year event at 3 ft SLR. At 2 and 3 ft SLR, the drainage system is especially sensitive to the 100-yr event, seeing the greatest increase in deep flooding compared to the 5- and 10-year events.

### Indicators of corrosive environment in storm drains

**Fig. 6:**
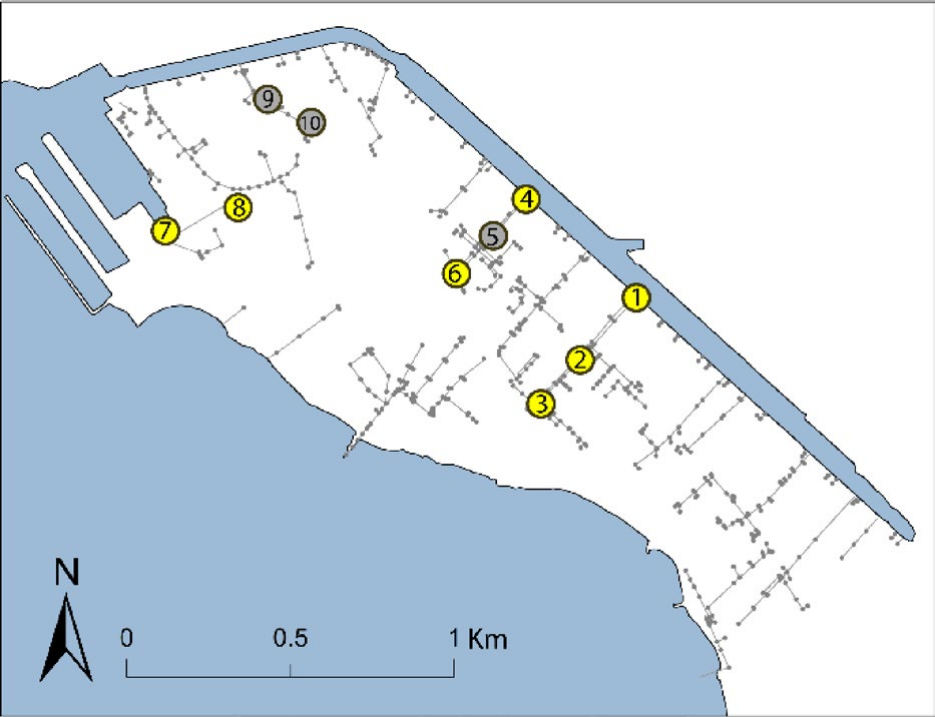
Locations of storm drainage monitoring sites recording water depth, temperature, and conductivity.

High salinity and warm temperatures may accelerate corrosion of storm drainage materials such as concrete and steel rebar. Salinity exposure was quantified as mean and maximum percent seawater measured at each storm drain monitoring site (Fig. 6, Table 2). For this analysis, 100% seawater is assumed to be 35 ppt. Mean percent seawater is above 50% for all but one monitoring site and maximum percent seawater exceeds 88% for all sites. The lowest maximum salinities occur at the two sites furthest from an outfall, while highest maximum salinity occurs at a site observed to have very shallow water levels, indicating potential for concentration of salinity due to evaporation.

**Table 2:** Mean and maximum percent seawater and temperature (°C) at each storm drainage monitoring site.

Location	Mean % Seawater	Max % Seawater	Mean Temp	Max Temp
1	69.4	93.8	26.1	30.2
2	56.2	96.7	26.3	30.3
3	69.6	89.1	25.3	27.8
4	60.2	91.7	26.0	30.8
6	48.0	88.3	26.2	29.5
7	78.6	95.9	24.4	27.0
8	52.9	109.7	25.9	29.5
Average	78.6	95.9	26.1	30.2

The maximum recorded ocean temperature during our sampling period was 27.7 °C at the NOAA Honolulu tide station (station ID: 1612340)^12^. In our data, maximum recorded water temperatures exceed local oceanic maximums by over 3 °C. Evidence of tidal influence on water levels was observed in every monitoring location, indicating that storm drainage pipes are exposed to wetting–drying conditions. Sites 5 and 9 were excluded due to suspected freshwater dumping and inconsistent water levels, while Site 10 was omitted due to insufficient data caused by instrument malfunction.

## Discussion

### Concerning vulnerabilities for Waikīkī

In Waikīkī, PCSWMM model simulations show that gravity drainage will progressively fail as sea level continues to rise, indicating a need for solutions to either prevent backflow or adapt to living in inundated areas. Figures 4d and 5 indicate outfall capacity is already severely limited today (stage 1 failure), and our simulations project that all outfalls will be submerged at and beyond 2 ft SLR. Figure 4a,b indicates precipitation events can generate inlet flooding significantly earlier than projected based on SLR alone. At present sea level, a 5-year precipitation event can cause as many inlets to backflow (≥ 0.5 ft) as 3 ft SLR, while a 100-year precipitation event produces flood depths sufficient to stall emergency vehicles (≥ 2 ft) at nearly one-third of inlets (stage 2 failure). At 2 ft SLR, over two-thirds of the pipe system will be full of water (stage 2 failure), further reducing drainage capacity. At 3 ft SLR without any precipitation, significant flooding begins to occur at inlets (stage 3 failure) with 17% of inlets with shallow flooding (≥ 0.5 ft). At 4 ft SLR, 95% of pipes are full of water during sunny conditions, making the area vulnerable to significant flooding even during minor precipitation events, as there is minimal capacity for stormwater drainage. Additionally, at 4 ft SLR, over half of all inlets reach the shallow flood threshold, indicating hazardous conditions for small vehicles. Western Waikīkī and Saratoga Road experience inlet flooding sooner than eastern Waikīkī, which aligns with previous modeling in this area^52^. Figure 4b indicates the number of additional inlets flooded by the 100-year event compared to by SLR alone is greater at 1 and 2 ft SLR compared to 3 and 4 ft SLR. Since SLR alone generates flooding at few inlets at 1 and 2 ft SLR, there is a large increase in total number of inlets flooded when the 100-year event occurs. However, since at 3 and 4 ft SLR, tidal conditions are already generating flooding at a significant number of inlets, the additional number of inlets that flood during the 100-year event is smaller.

Stage 4 failure occurs when SLR flooding is compounded by precipitation events. Figures 4d and 5 indicate, when accounting for precipitation, 100% of outfalls will be plugged and over 85% of the pipe system will be full at and beyond 1 ft SLR. While shallow flooding occurs at less than 1% of inlets at 1 and 2 ft SLR without precipitation, adding precipitation results in more shallow flooding than occurs at 4 ft SLR alone. Figure 4c indicates at 4 ft SLR, sea level surpasses precipitation as the dominant driver of flooding, indicating that gravity-driven storm drainage is no longer functional for over half of the system. Even a 5-year event at 4 ft SLR will cause 95% of inlets to reach the shallow flood threshold and 82% of inlets to reach the deep flood threshold.

Published exceedance probability levels for Honolulu (station ID: 1612340)^12^, indicate that a 1% chance elevated marine water level will raise local sea level 1.4 feet above MHHW, flooding an area roughly equivalent to the FEMA pluvial 1% floodplain. The Hawaii State Climate Commission recommends the 1% high water level as minimum base flood elevation for building to allow sufficient freeboard during elevated waters^75^. Future work should incorporate these thresholds to account for extreme compound flood conditions.

**Fig. 7:**
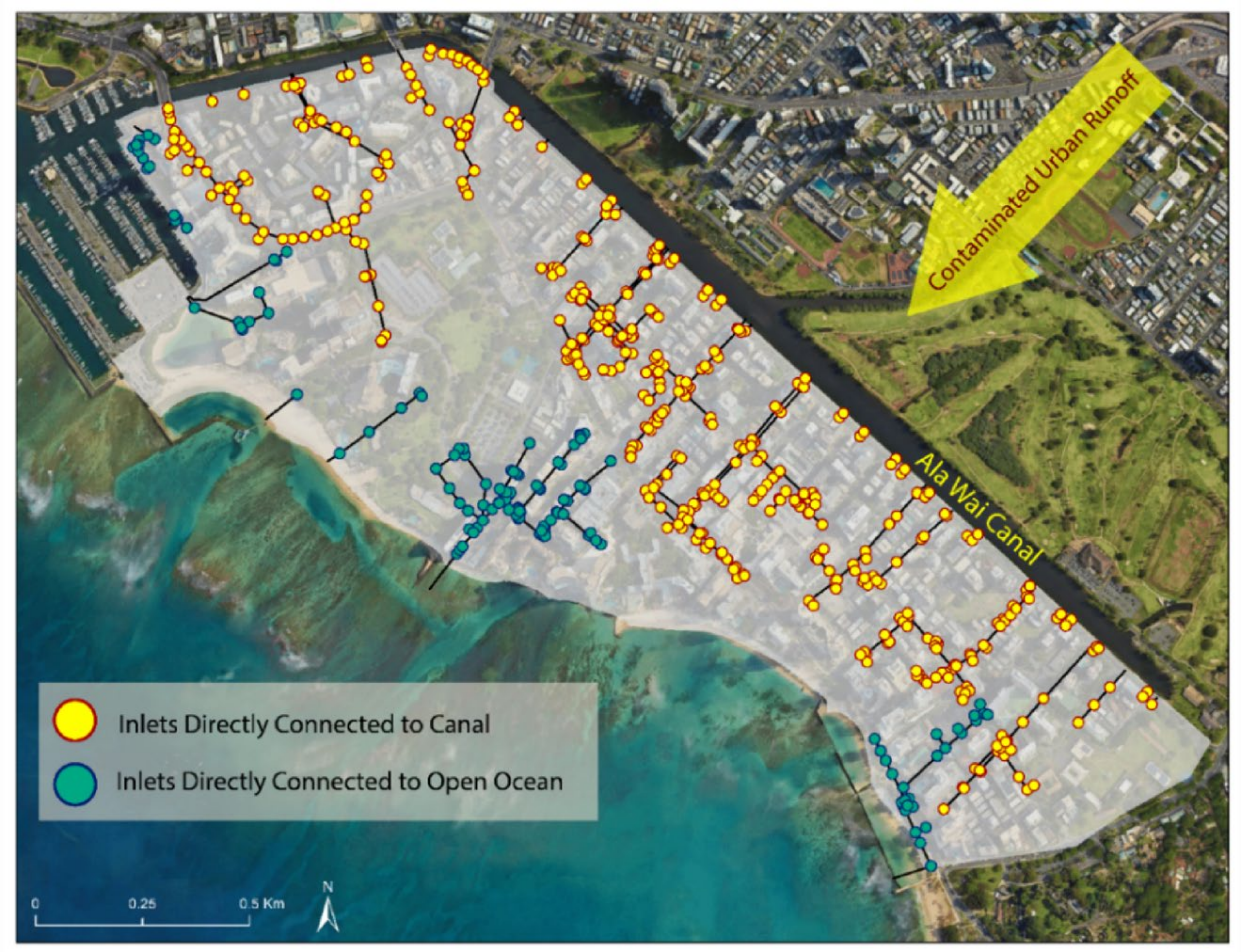
Polluted surficial and subsurface runoff from the urban drainage area of the Ala Wai Canal has led to high levels of contamination in the canal water. This image highlights drainage inlets (yellow) with direct connection to the canal, which are potential sources where contaminated water can make its way to the surface, posing a health concern. The software used to generate the map in this figure is Esri ArcMap version 10.8.2 https://www.arcgis.com/, Google Earth Pro version 7.3 https://earth.google.com/, and Adobe Illustrator version 28 https://www.adobe.com/products/illustrator.html.

Storm drainage backflow in Waikīkī presents not only hazardous driving conditions but also a threat to human health. During precipitation events, urban runoff from the heavily populated Ala Wai watershed flushes into the Ala Wai Canal. Contaminants can also enter the canal through groundwater containing cesspool and sewage leak effluent; with concentrations correlating to peak tourism season^78,79^. Backflow observed at Waikīkī storm drains observed during present-day precipitation and king tide conditions provides a direct pathway for contaminated water from the Ala Wai Canal to come into contact with the public and infrastructure^53^. Our monitoring results demonstrate that the majority of standing water in the Waikīkī storm drainage system originates from the Ala Wai Canal. Since the base water level in the drainage system is influenced by sea level and over 75% of the system is connected to the heavily contaminated canal, these conditions will likely worsen with SLR (Fig. 7).

### Management implications

This study highlights the importance of understanding the temporal thresholds at which tidal levels will significantly reduce effectiveness of drainage systems to accommodate precipitation runoff both for management of floodwaters and contamination concerns. Figure 4c reveals implications for flood mitigation based on dominant drivers of shallow inlet flooding. At present and up to 2 ft SLR, precipitation events are the main driver of inlet flooding in Waikīkī. This points to the viability of short-term (present through 2050) management solutions that focus on mitigating rainfall runoff (i.e., green infrastructure, low impact development, bioswales, and permeable pavement) to reduce inlet flooding. At 3 ft SLR a tipping point is reached at which nearly 20% of simulated shallow inlet flooding is initiated by high tidal levels. At 4 ft SLR, high tidal levels become the dominant driver of two-thirds of shallow inlet flooding, indicating that gravity-flow drainage will become ineffective in draining large precipitation events, and longer-term management solutions targeting the years 2070 and beyond should focus on preventing backflow from tidal conditions at outfalls (i.e., pumping stations, check valves, tide gates, and elevated outfalls). Formulation of design guidelines for coastal storm drainage that consider temporal thresholds in Honolulu could establish a best-practice for infrastructure planning that optimizes drainage performance in the face of rising seas and intensifying precipitation. Strategies that focus on mitigating rainfall runoff should be prioritized through 2050, while strategies that focus on prevention of backflow should target implementation by 2070 and beyond.

The timing of flooding drivers has additional implications for planning related to public health. Figure 5 identifies precise locations of potential backflow of contaminated water in Waikīkī. As the source of contamination is the tidally-influenced water of the Ala Wai Canal, scenarios in which the driver of inlet flooding is predominantly tidal have potential for higher levels of contamination in floodwater. To date, limited action has been taken to address public health concerns associated with contaminated backflow in the Ala Wai Canal. To effectively mitigate these concerns, comprehensive efforts are needed to enhance water quality through improved watershed management aimed at reducing pollution inputs, especially at and beyond the 3 ft SLR scenario. Recommended strategies include the implementation of green infrastructure, permeable pavement, and other stormwater best management practices (BMPs), along with the enforcement of stricter regulations governing the disposal of waste materials into the natural environment.

Though this study was designed considering local conditions, the results of modeled scenarios may indicate similar conditions in other coastal cities. For example, Miami Beach has been experiencing tidal flooding and elevating roadways and re-routing drainage to pump stations to reduce pressure on the stormwater system^80^. The 2021 New York Stormwater Resilience plan concludes that flooding at outfalls without tide gates may cause backflow and upland flooding through street drains, and notes that the prolonged presence of saltwater can damage stormwater infrastructure^81^. Location-specific management in these and other coastal cities would be enhanced by understanding of the SLR thresholds at which coastal storm drainage will no longer effectively drain precipitation runoff.

The contamination concerns raised by this study are highly applicable to other densely urbanized areas. Like Honolulu, Shanghai faces challenges from its dense population, low elevation, shallow water table, impermeable surfaces, tidal canals, and aging storm drainage. The city’s US$21 billion stormwater masterplan aims to install green, blue, and grey infrastructure while maintaining existing drains by 2030^82^. Tokyo, a leader in flood management, plans to expand its sewer system, build communities on higher ground, double underground reservoir capacity, raise seawalls, and protect underground spaces with flood barriers^83^. In Mumbai, projected sea-level rise by 2050 could submerge 40 km2, exacerbating issues with its aging drainage system. Despite launching a project to double the drainage system’s capacity in 2005, progress remains incomplete due to financial and institutional barriers^84,85^. Rapid population growth, poorly functioning drainage infrastructure, and tidal flooding are impacting Ho Chi Minh City, prompting management strategies like dredging, infrastructure upgrades, tidal valves, and pump stations^86^. Understanding when and where tidal levels and precipitation events will trigger storm drainage failure should be considered to anticipate hyper-localized contamination concerns and develop planning efforts that target mitigation specific to the predominant driver of flooding.

### Comparison to hydrostatic models

Inundation of coastal areas due to rising sea levels is often simulated using hydrostatic, or “bathtub” modeling techniques. Such models use digital elevation models to identify areas located below projected sea levels^52,61,87–89^. Hydrostatic modeling is a valuable tool due to its low computational cost and ability to generate visual representations of potential inundation useful for adaptation planning. Notable applications include the NOAA SLR Viewer and the Pacific Islands Ocean Observing System State of Hawaiʻi SLR Viewer^90,91^.

However, this modeling approach solely considers elevation and does not account for precipitation or drainage systems. Flood inundation maps should also consider the role of stormwater infrastructure, which alters the ability of a coastal area to drain and accommodate inputs from precipitation. As compared to the hydrostatic model from Habel et al. (2020), which simulates passive flooding from static SLR, the PCSWMM model identifies a similar number of flooded inlets per increment of SLR. However, as the hydrostatic model does not consider precipitation, when we account for precipitation at present sea level as compared to passive modeling at present sea level, the PCSWMM model identifies 22% more flooded inlets during a 5-year event, 34% more flooded inlets during a 10-year event, and 50% more flooded inlets during a 100-year event (flooding at shallow threshold). This comparison shows that while hydrostatic modeling is effective for projecting passive flooding in heavily urbanized coastal areas, dynamic modeling is necessary to accurately project flooding during precipitation events.

### Corrosion potential

In the subsurface, the interaction between salinized water and the pore spaces in concrete can lead to crystalline corrosion, characterized by expansive cracks^29,92^. Chloride-induced corrosion of reinforcement has been recognized as a major factor in reducing the service life of reinforced concrete structures^93,94^. A tidal, wetting–drying environment, like that of the Waikīkī subsurface, is considered one of the harshest for infrastructure^95^, and water intrusion through defects in the local sewer system has been identified^96^. We measured salinity levels in the Waikīkī storm drainage system that are likely to exacerbate corrosion, consistent with findings from similar studies in the area^79^. Furthermore, measured water temperatures frequently exceeded local oceanic maximums. As temperature is known to increase reaction rates, future climate conditions may increase corrosive potential of infrastructure^97^**.**

The City & County of Honolulu standard specifications for public works standards were established in 1986^98^. Given that various factors can affect the structural integrity of concrete, a localized study is necessary to update these specifications to reflect modern conditions and concrete compositions^99^. Remediation efforts have already begun in some areas of Waikīkī to replace corroded culverts. The Honolulu Department of Facilities Maintenance presently lacks the resources to conduct a complete assessment of drainage defects but plans to do so in the future using CCTV technology. Future design standards for more climate-resilient belowground infrastructure could consider a) *structural changes* such as i) increasing concrete thickness, ii) reducing water content in cement to minimize pore space, and iii) adding fillers to enhance structural integrity under pressure; and b) *alternative materials* such as i) fiberglass or carbon fiber rebar and ii) corrosion-resistant concrete coatings and additives^100^.

## Summary and conclusion

The impact of climate change on drainage infrastructure capacity poses a hidden threat to public health and safety. Rising sea levels, combined with high tides and extreme precipitation events, increase the risk of both chronic and event-based flooding in coastal urban areas. In this study, a robust PCSWMM model simulates the effects of precipitation (5-, 10-, and 100-year events) and sea-level rise (up to 4 feet above mean higher high water) on a coastal urban drainage system. We demonstrate how water from the heavily contaminated Ala Wai Canal is entering Waikīkī, the main tourism hub of the State, and project that this issue will occur more frequently and with greater magnitude over time. Major findings for Waikīkī include:

- 71% of outfalls are submerged at present MHHW and 100% of outfalls will be submerged by MHHW + 2 ft SLR.
- Precipitation can generate flooding significantly earlier than based on SLR alone. A 5-year event at present sea level floods an additional 5% of inlets compared to MHHW + 3 ft SLR, while a 10-year event floods twice as many inlets as MHHW + 3 ft SLR (shallow flood threshold).
- By 2050 (2 ft SLR, projected under intermediate high scenario), a 5-year precipitation event could cause flooding severe enough to disrupt transportation and contaminate stormwater inlets across 71% of Waikīkī.
- Precipitation is the dominant driver of inlet flooding through 2 ft SLR, while at 4 ft SLR the dominant driver becomes backflow from high tidal levels. This validates the utility of an adaptive management approach that targets reduction of rainfall runoff through 2050, phasing in measures to reduce tidal backflow by 2070 and beyond.
- As tidal waters become the dominant driver of inlet flooding at and beyond 4 ft SLR, there is risk of contamination present in flood waters at over 75% of drainage system inlets.
- Our results find a similar number of flooded inlets per increment of sea-level rise as do hydrostatic model results. Our results indicate 22% more flooded inlets during a 5-year event, 34% more flooded inlets during a 10-year event, and 50% more flooded inlets during a 100-year event than the hydrostatic model at present sea level.
- Tidally-influenced water levels and salinity were observed at every storm drain monitoring site. Measured water temperatures in the storm drains frequently exceeded local oceanic maximums.

This study highlights the importance of incorporating precipitation and drainage infrastructure into coastal compound flood models to better understand how drivers of coastal flooding change over time. We validate the hydrostatic modeling method for projecting sea-level rise flooding in coastal urban areas and emphasize the need for more dynamic modeling to account for precipitation. Salinity and water temperature measurements in storm drains indicate significant corrosion risks, likely worsening drainage issues. To address these concerns, drainage backflow and corrosion under sea-level rise and precipitation scenarios should be included in risk assessments and resiliency planning for Waikīkī and other coastal urban areas.

## Methods

The Stormwater Management Model (SWMM5), developed by the EPA in 1971, is a dynamic rainfall-runoff simulation model that consists of semi-distributed hydrology, 1D hydraulics, and is capable of event-based and long-term simulations of precipitation^101^. SWMM5 has been used globally for a variety of applications related to stormwater infrastructure and urban planning and is a FEMA-approved model for National Flood Insurance Program Studies^102–104^. With rapid processing power, the model can represent a storm drainage system with a high level of detail at an unlimited system size, including variable open and closed pipe shapes. Relevant SWMM5 capabilities for this study include user-defined inflows, time-varying precipitation, evaporation of surface water, infiltration of precipitation into unsaturated soils, interactions between groundwater and the drainage system, and nonlinear reservoir overland flow routing. The model can simulate backwater effects, surcharging, flow reversals, and ponding. Spatial variability is accounted for by dividing the study area into a collection of homogeneous subcatchments which receive precipitation and generate runoff based on percent pervious and impervious sub-areas. Overland flow can be routed between sub-areas, subcatchments, and entry points of a drainage system. SWMM5 uses a 1-dimensional scheme with Manning’s equation to calculate overland flow and the Green-Ampt method to simulate infiltration^101^. The full dynamic wave form of the 1D St. Venant equation is used to estimate rainfall and both closed-pipe and open-channel flow routing.

The Personal Computer Storm Water Management Model (PCSWMM) is a dynamic model for simulating rainfall-runoff for a single event or long-term period in urban areas^74^. PCSWMM was developed by Computational Hydraulics Inc. as a spatial decision support system for SWMM5 for modeling stormwater, wastewater, watershed and water distribution systems. PCSWMM expands on the capabilities of SWMM5 to include GIS and time series management in a user-friendly interface^74^. The software has been used widely for planning, design, and analysis of drainage systems, and is ideal for modeling urban catchments with fast responses^62,67,105–108^.

### Model configuration

To configure the PCSWMM 1D domain for our study site, we used a 2-m horizontal resolution digital surface model (DSM) developed by synthesizing available elevation data, United States Geological Survey developed breaklines, and hand-edited correction. This process addressed interpolation artifacts, data gaps (holidays) and misclassification errors (such as LiDAR misclassification and sparse data interpolation), which could lead to model errors. The PCSWMM Watershed Delineation Tool was used to generate subcatchments for the study area based on the DSM. Percent impervious surface for each subcatchment was derived using the NOAA Coastal Change Analysis Program 2006 Regional Land Cover Dataset^109^. Infiltration parameters were assigned to each subcatchment using soil data from the United States Department of Agriculture Natural Resources Conservation Service Web Soil Survey^110^. O’ahu is served by a Municipal Separate Storm Sewer system (MS4). Storm drainage system properties were derived from GIS data, Civil Drainage Maps, and As-built documents provided by the City and County of Honolulu, Department of Planning and Permitting, Honolulu Land Information System^49^. Attributes provided for the drainage system include the geographical location of inlets, pipes, and outfalls, cross-sectional shape and area of pipes, pipe length and material, and invert elevations of inlets and outfalls. Locations and invert elevations for ~ 800 inlets and outfalls were verified as part of this study with field measurements conducted in Summer, 2023. The stormwater system was simplified where applicable, removing unconnected inlets or pipes and removing closed loops. Roughness values for the pipe materials were acquired from the 2017 Department of Planning and Permitting City and County of Honolulu Storm Drainage Standards document^111^ Hourly precipitation data was acquired from the NOAA National Centers for Environmental Information Local Climatological Data Station at the Honolulu International Airport^112^. Hourly sea level was acquired from the NOAA Honolulu tide gage (station ID: 1612340)^12^.

### Observational data

To collect quantitative, observational water level data for calibration and validation, 10 Solinst LTC 5 Levelogger pressure transducers (here referred to as transducers) were installed throughout the Waikīkī storm drainage system (Fig. 6). The transducers were individually calibrated considering conductivity, following the Solinst Levelogger Software 4.6.2 protocol^113^. Transducers record absolute pressure, which can be converted into water depth using the fluid statics equation for pressure^114^. To convert the absolute pressure readings into water level data, the pressure readings are compensated with atmospheric pressure recorded by a Solinst Barologger.

The elevation of the transducer and rim elevation of the storm drainage inlet where each transducer was deployed were surveyed with an RTK GPS with respect to local mean sea level (LMSL; Datums – NOAA Tides and Currents, present epoch: 1983–2001). Two transducers were installed at outfalls along the Ala Wai Canal and one transducer was installed at an outfall along the Ala Wai Boat Harbor. The remaining transducers were installed in the bottom of storm drainage inlets throughout the study area. Water depth data was collected in 5-min increments from February, 2023 to May 2024.

In addition to water levels, the deployed transducers record conductivity and temperature, providing insights into the corrosive environment within the storm drains. To investigate trends in conductivity and temperature in the storm drainage system we calculated average and maximum conductivity and temperature recorded from February 2023 – March 2024. The Levelogger 5 uses automatic temperature compensation to normalize conductivity to specific conductance at 25 °C. We use the Python implementation of the Thermodynamic Equation of Seawater to derive absolute salinity (mass fraction of salt in seawater) from conductivity^115^.

### Calibration

Using rainfall intensity duration values published for the Ala Wai watershed, we identified a 2-year recurrence interval precipitation event on April 2nd, 2023 which generated 2.8 in. of precipitation in 3 h^42^. To model this scenario, data collected by the three transducers at outfalls were assigned as tidal boundary conditions for all nearby outfalls, and the model precipitation gage was populated with hourly data from the Honolulu International Airport^112^. Local rainfall data is not presently being collected and published for the Waikīkī area, so we selected the Honolulu Airport as the best candidate for a nearby weather station as it is located within similar proximity from the coastline and 11 km northwest of Waikīkī. Both the Honolulu Airport and Waikīkī are oriented Southeast and are exposed to Kona low pressure systems which typically bring the most severe precipitation along this coastline, supporting the assumption that a similar amount of precipitation occurred at the Honolulu Airport and Waikīkī during the calibration and validation events. However, use of hourly rainfall data from a station outside of the study area introduces potential variation between the data and true rainfall conditions in the study area. A future study might consider installing a local weather monitoring station to provide more accurate and high-resolution rainfall data.

**Fig. 8:**
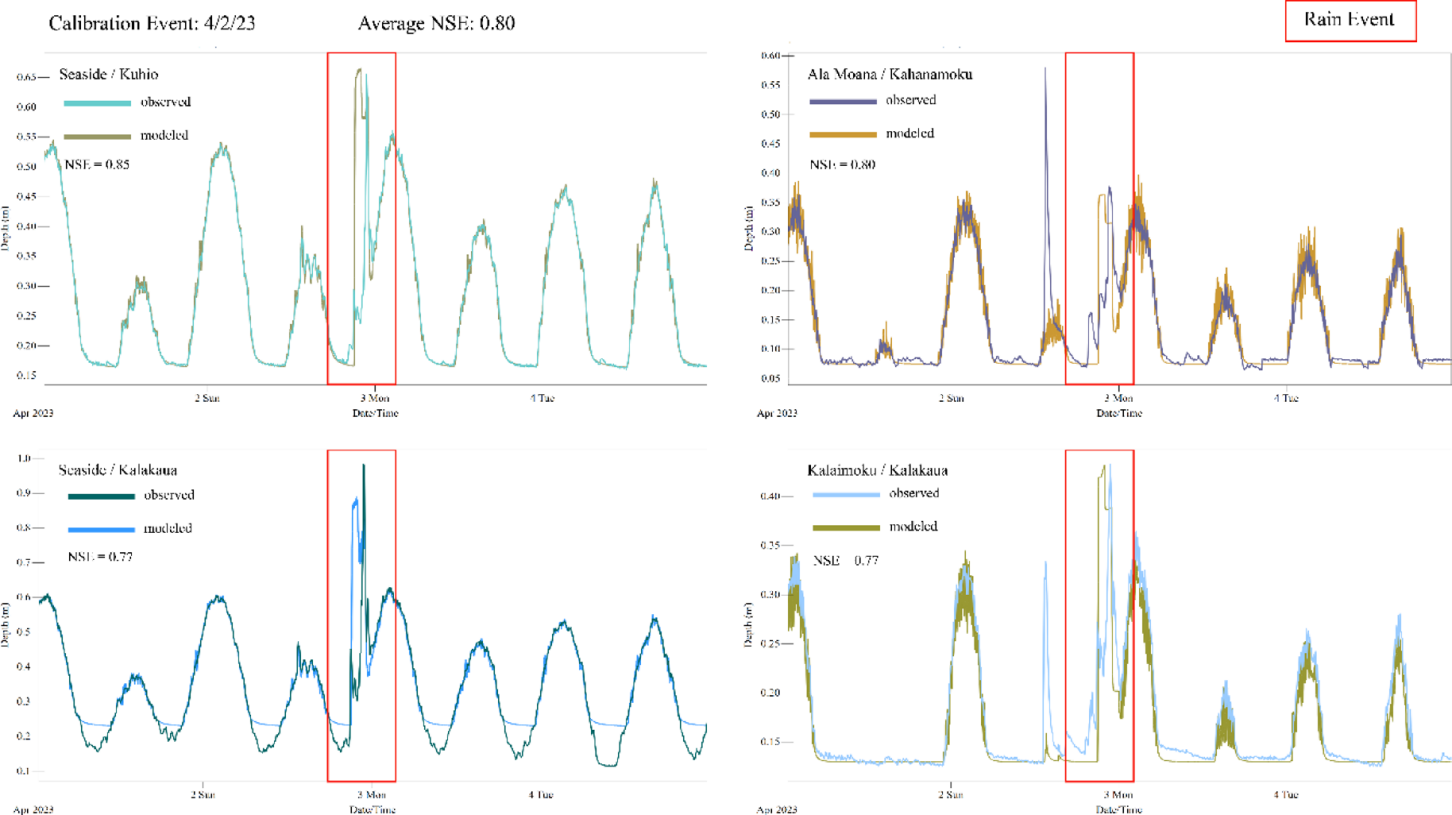
Observed and modeled output of water levels for the calibration precipitation event; April 2023. Observed water levels were measured by pressure transducers installed in the bottom of storm drain inlets.

**Fig. 9:**
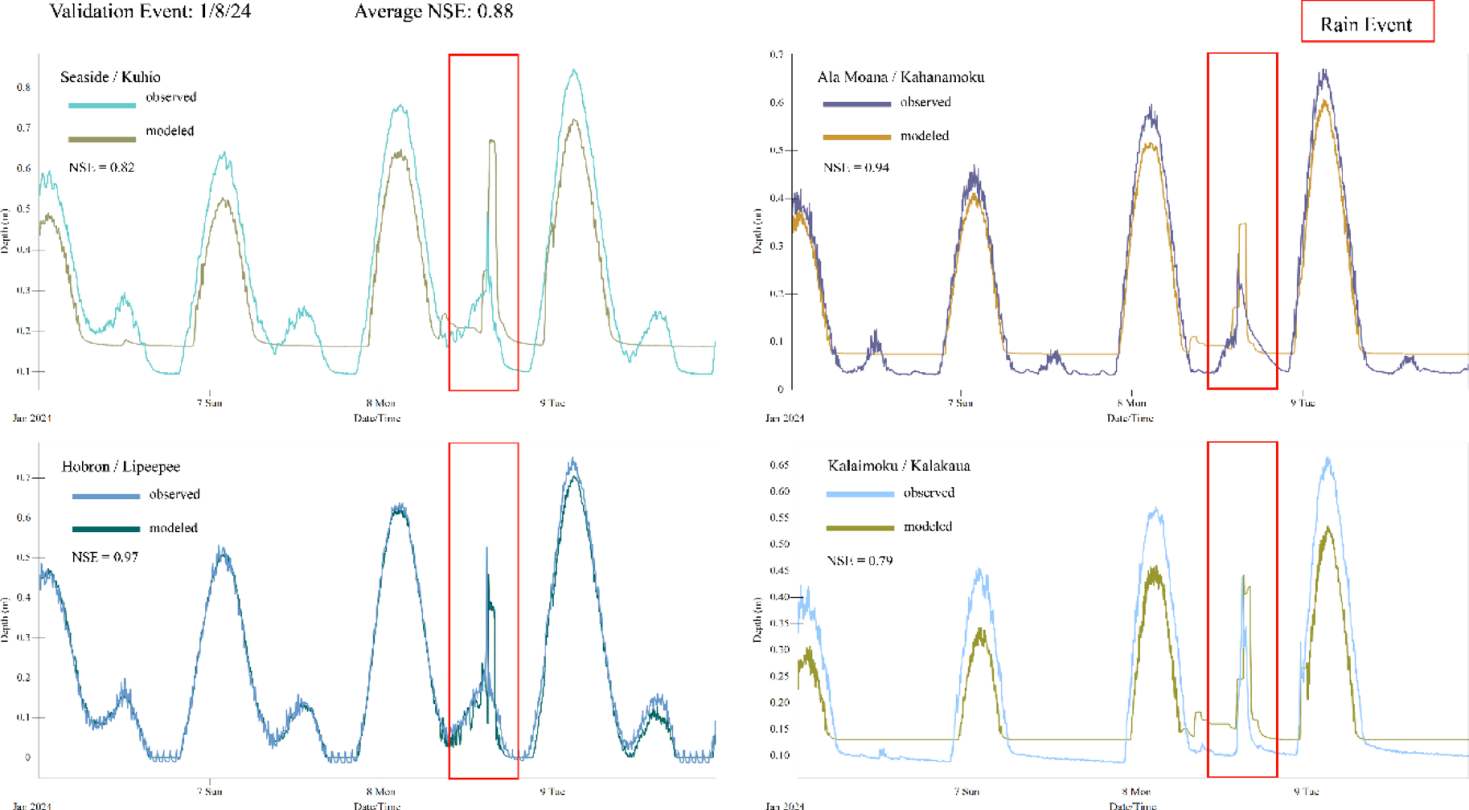
Observed and modeled output of water levels for the validation precipitation event; January 2024. Observed water levels were measured by pressure transducers installed in the bottom of storm drain inlets.

The PCSWMM Sensitivity-based Radio Tuning Calibration tool was used to assign each parameter for the subcatchments, inlets, outfalls, and pipes layers an uncertainty as a percentage of the present, best-estimated value. This value was used to calculate the uncertainty range for each parameter. Sensitivity testing involved adjusting this range for each parameter and observing the effect on modeled goodness of fit. Inlet or outfall invert elevations and subcatchment outlet assignments were found to be the most sensitive parameters and were manually adjusted to align modeled tide and precipitation peaks with observations from four transducer locations (Figs. 8, 9). After conducting a field survey of invert elevations for ~ 800 inlets and outfalls throughout the study area, the GIS layer provided by the City and County of Honolulu was found to contain data gaps and outdated values for some invert elevations, identifying this parameter was as a likely source of error. Furthermore, adjusting inlet invert elevations had the largest effect on modeled tidal conditions, so was selected as a calibration parameter. Subcatchments were delineated based on the DSM, but the PCSWMM software allows each subcatchment to route to only one inlet. Adjusting the drainage inlet for subcatchments had the largest effect on modeled precipitation conditions, so was selected as the second calibration parameter.

Nash–Sutcliffe efficiency (NSE) quantifies the degree of agreement between simulated and observed data. NSE range extends from − ∞ to 1, where values > 0.5 are generally deemed satisfactory with NSE = 1 representing an ideal match^116^. After manual parameter tuning, modeled data as compared to transducer data have an average NSE of 0.80. To validate our model, we ran a second simulation corresponding with a 2-year recurrence interval precipitation event on January 8^th^, 2024, which generated 1.41 in. of precipitation in 1 h^42^. Water level observations from this event at four transducer locations were compared to model output, with an average NSE of 0.88. We note as a limitation of this methodology that though the largest precipitation events that occurred during our data collection were used for calibration of the model, those events are smaller than the simulated precipitation events in our analysis.

### Vertical uncertainty

The DSM used in this study has a horizontal resolution of 2 m, and a vertical uncertainty quantified as the 95% confidence interval around the mean (± 30 cm). The average uncertainty of our survey data is ± 5 cm and the linear error, or the error at the 95% confidence level, is ± 10 cm. We assume ± 10 cm uncertainty for hand-collected vertical measurements of invert elevation due to potential for sediment buildup in inlets or deep catchment basins over- or underrepresenting elevation. Uncertainty of the pressure transducer is ± 0.15 cm^117^. Uncertainty of the water level peak in the Ala Wai associated with precipitation events is ± 8.73 cm. Average RMSE for the validated model is 5.8 cm. Sources of error are summed in quadrature (the square root of the sum of the squares) to calculate a total vertical uncertainty of 33.31 cm.

A surface area of 2m^2^ is assigned to each inlet to allow for flood volume storage, from which flood depth is calculated. Though a small floodable area has potential to overestimate flood depth, this size was selected for this study to ensure all potential flood-prone locations are identified. Authors recommend that localized flood studies are conducted to improve uncertainty in flood depth. The findings presented primarily serve to identify locations of most severe future flooding for this study area and alert to potential effects of minor and major floodwaters generated during conditions of rainfall and sea-level rise.

### Quantifying storm drainage performance

Model simulations are allowed a spin-up period before reporting of results to remove instabilities associated with initial conditions. Model output includes a value for average and maximum water depth for each simulation. To represent the most consistent water level and avoid model instabilities, average water depth is considered in static sea level scenarios. To capture peak water levels during precipitation scenarios, maximum water depth is considered in precipitation scenarios. To compute depth of water above an outfall or inlet, the appropriate water depth value is subtracted from the height of the drainage structure, which is the distance between its invert elevation and surface elevation, with negative values indicating flood depth. Inlets are evaluated for shallow flooding when water depth is 0.5 ft or more above the inlet surface elevation and deep flooding when water depth is 2 ft or more above the inlet surface elevation to align with previously used benchmarks^52,77^. Outfalls are classified as plugged when the maximum depth of water in the simulation exceeds the depth of the outfall by greater than 5 cm. For the analysis of pipes full, model output includes a binary value indicating the maximum simulated water level in the pipe reached the depth of the pipe at any point in the simulation. To compute percentage of pipe system full, the length of all full pipes is divided by the total length of all pipes in the study area.

### Climate change scenarios

In Honolulu, sea levels are expected to rise approximately 1 foot by 2040, 2 feet by 2050, 4 feet by 2070, and 6 feet by 2100 according to the “intermediate high” scenario from the U.S. Interagency Sea Level Rise Task Force^76^. For this study, we simulate present sea level, using the mean higher high water (MHHW) datum of local mean sea level + 1.08 ft as our baseline of present sea level, and add 1-foot increments of sea-level rise up to MHHW + 4 ft (5.08 ft). To account for groundwater rise, the PCSWMM Aquifer Editor was used to assign properties to subcatchments based on their soil layer classification. Groundwater table elevation was assigned as a fixed level equal to sea level at each scenario.

MHHW was selected as a base level in this study to align with the methodology of previous work ^15,52,76^. The Hawaiʻi State Sea Level Rise Viewer also uses a baseline of MHHW for its projections and this data is incorporated into planning documents for the City and County of Honolulu such as the 2018 Sea Level Rise Guidance document. Future work might consider modeling simulations at multiple tidal datums to provide a more complete suite of potential flood conditions, as well as computing the probability of MHHW and a precipitation event coinciding. To generate 5-year, 10-year, and 100-year precipitation simulations, a hyetograph was generated based on the Rainfall Intensity Duration values calculated by the US Army Corps of Engineers for the Ala Wai Watershed as part of the Ala Wai Flood Risk Management Project^42^. The data from this report was determined from maps and nomographs in Giambelluca and others, 1984, DLNR. The intensity duration values are inputted into the PCSWMM Design Storm Creator which uses the Chicago method to generate a precipitation event based on a 24-h duration. Our transducer data from outfall locations show that a precipitation event causes a spike in water level in the Ala Wai Canal. For simulated precipitation events, a peak in water level in the Ala Wai canal is estimated as a function of precipitation volume. Pressure transducer data was collected by C. Obara for one precipitation event in April 2023 and by S. Spengler for six precipitation events from December 2020 – January 2021 at the storm drain outfall at Seaside Ave. Maximum height of Ala Wai Canal above base level was plotted against total rain volume acquired from the NOAA precipitation data from the Honolulu International Airport station to derive an equation for estimating the peak increase in water height given a total rain volume. The water level peak is distributed proportionally to the hyetograph derived by the Chicago Method for the 24-h design storm, and the water level peak is offset 1 h after the hyetograph peak to account for the lag in drainage from the Mānoa, Pālolo, and Makīkī valleys. Via this methodology, we superimpose an estimated water level increase corresponding to each precipitation event onto the tidal boundary condition for outfalls along the Ala Wai Canal as follows: 0.67 m for the 5-year event, 0.81 m for the 10-year event, and 1.29 m for the 100-year event. A 24-h design storm represents a simplified precipitation event and is not representative of all possible rainfall conditions or storm durations. Future studies might consider simulations that include storms of varying durations.

## Data Availability

The data that support the findings of this study are available from the corresponding author, C.O., upon request.
